# Our Board of Censors

**Published:** 1893-12

**Authors:** C. D. Hurt


### OUR BOARD OF CENSORS.

There is an old adage : " Anything worth being done, should be well done. " It is as true as old. I wish to call attention to the Board of Censors of the Georgia Medical Association to some infractions upon our code of ethics, which tend to make lukewarm some of the best members of the Association. Underlying the correct principles of this code there ought to be a spirit of fairness and right doing which will keep each man ready in his feelings to do as he would be done by.

There are a few influences which prompt men to participate in these violations after solemnly agreeing that they will not.

First. That part of our nature which loves eclat, the praise of men.

Second. A desire to outstrip our neighbor, trusting to our natural and acquired endowments which win popularity for us, we flaunt our names and auto-biographies and cuts of our physiogamies in the secular newspapers, supposing that our fame will justly entitle us to this liberty with our brethren.

Third. A laudable ambition sometimes becomes morbid in its growth, and like a selfish monarch it dominates judgement, charity, kindness, &c., until a clever gentleman is transformed into a peevish, snarlish, selfish autocrat.

Fourth. Men are thus led on to a forgetfulness of those higher, dignified and elevating principles of our code and step down into the quagmire which propagates and nurtures Charlatans, imposters and pretenders &c. What rejoicing in this latter class when a good man in our profession folds his hands, turns traitor to science, and joins with them to deepen the cesspools of corruption.

Fifth. Men sometimes forget that two wrongs will not make a right, and are provoked into this mode of advertising to the public to check and counteract the same evil in others.

Whatever induces man to depart from our code of ethics, violate its principles, transgress the rules of propriety in seeking unfairly the advantage of his neighbor ; the ultimate result to him is surely self destruction. Like a bubble he may be lifted above the heads of giants in science and intellect to burst and disappear as vapor, or, suffer the treachorous phantom balloon to take its victim to an altitude which guarantees destruction, precipitate him to death by the fall. Practically, what is the difference between a respectable, high-toned, clever doctor, a member of the Georgia Medical Association, who allows some reporter to interview him, take his likeness with a kodack, give him a front page, a picture and a column or two of write up, just for his cleverness and prominenee, and the travelling mountebank charlatan who orders these displays and whatever else he wants and pays the publisher for them ? I leave the question with you. What is the difference between the very flattering accounts given in our newspapers of operations done at the hospitals and elsewhere by Dr. ——— which certainly tend to advertise him contrary to the Code of Ethics ; and a man who comes to our city for a short stay, rents an office, runs a standing advertisement in the newspapers in which he displays and recites his surgery and practice? Some of these violations may be chargeable to the energy of the newspaper reporter. Some may be the result of a desire on the part of certain men to increase their business. Whether from the one or the other cause, it is within the power of any man to prevent these frequent infractions upon the Code of Ethics.

What is the difference in the publication of a long interview with some intelligent layman, of all the salient points in the treatment adopted by some celebrity in his case and winding up by saying that Dr. A. & B., it is understood, will try this method and revolutionize practice in our city, and the proprietor of a patent medicine who culls some thrilling incident from a dime novel and winds up by reciting his wonderful discoveries and lauding himself and the favors of his nostrums ? I leave the question with you. Truly, what is sauce to the goose should be sauce to the gander, and what is right applied to any one member of an association is right to all its members.

If the dean of a reputable Medical college can suffer his name thus handled by the secular papers, why, for the sake of consistency, does he not teach his students to do likewise? If a professor decries such a course in charlatans and patent medicine venders, why for the sake of fairness does he not put a frown of unmistakable disapproval on any and all members who are guiity of such treason and trickery.

Then, and not till then, will our Medical Association work harmoniously, prosper legitimately and pull together in the development of science, and strive for that righteous emulation as to who can best work and best agree.

Let the Board of Censors faithfully discharge their duties by looking into any violation and deterring any member from varying from the course of rectitude. Let our code be dignified and by so doing we will bring honor and dignity to ousselves.

C. D. HURT.

